# Supplementary material for: Leveraging spatial transcriptomics data to recover cell locations in single-cell RNA-seq with CeLEry
Source: Nat Commun. 2023 Jul 8;14:4050. doi: 10.1038/s41467-023-39895-3 (PMC10329686; doi:10.1038/s41467-023-39895-3)

SUPPLEMENTARY INFORMATION

**Leveraging spatial transcriptomics data to recover cell locations in single-cell RNA-seq with CeLEry**

Qihuang Zhang[1]*, Shunzhou Jiang[2], Amelia Schroeder[2], Jian Hu[3], Kejie Li[4], Baohong Zhang[4], David Dai[5], Edward B. Lee[5],

Rui Xiao[2], Mingyao Li[2]*

These authors contributed equally: Qihuang Zhang, Shunzhou Jiang, Amelia Schroeder

* Correspondance:

Dr. Qihuang Zhang, qihuang.zhang@mcgill.ca

Dr. Mingyao Li, mingyao@pennmedicine.upenn.edu

**Supplementary Fig. 1.** Spotwise mean and standard error of normalized and log-transformed gene expressions for the six randomly selected gene clusters obtained from the mouse posterior brain data.

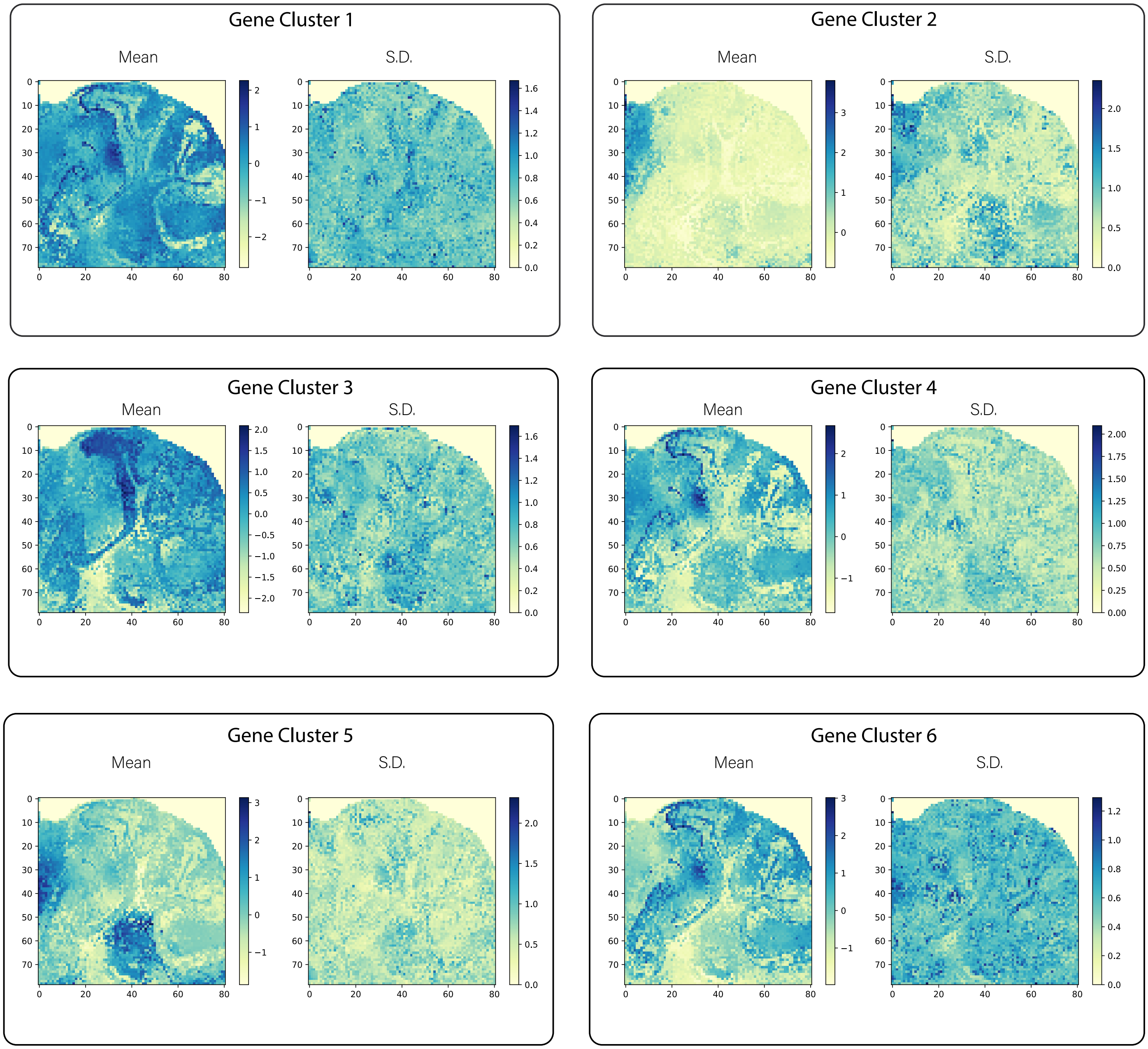

**Supplementary Fig. 2.** Additional examples of the gene map replicates obtained from performing data augmentation procedure to the mouse posterior brain data. Each gene was randomly selected from each gene cluster shown in Fig. 2a and the gene expression was normalized and log transformed.

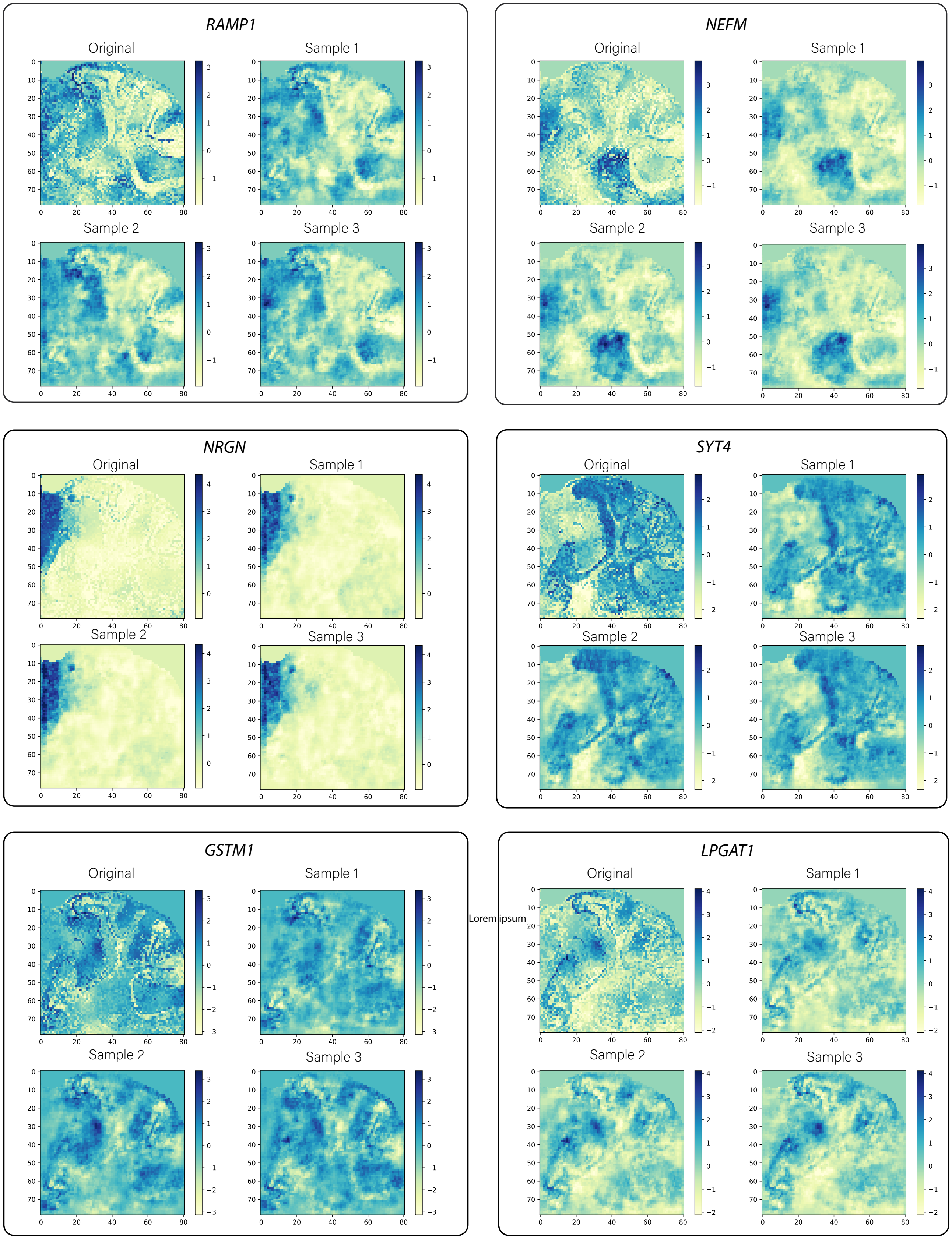

**Supplementary Fig. 3.** Gene expression map comparison between the biological replicates (tissue ID 151674, 151675 and 151676) and the artificial replicates produced by the data augmentation procedure. We randomly selected three genes (a) *CAMK2N1*, (b) *TNSB19*, and (c) *HPCA* from the LIBD human DLPFC data as examples. The gene expression was normalized and log transformed.

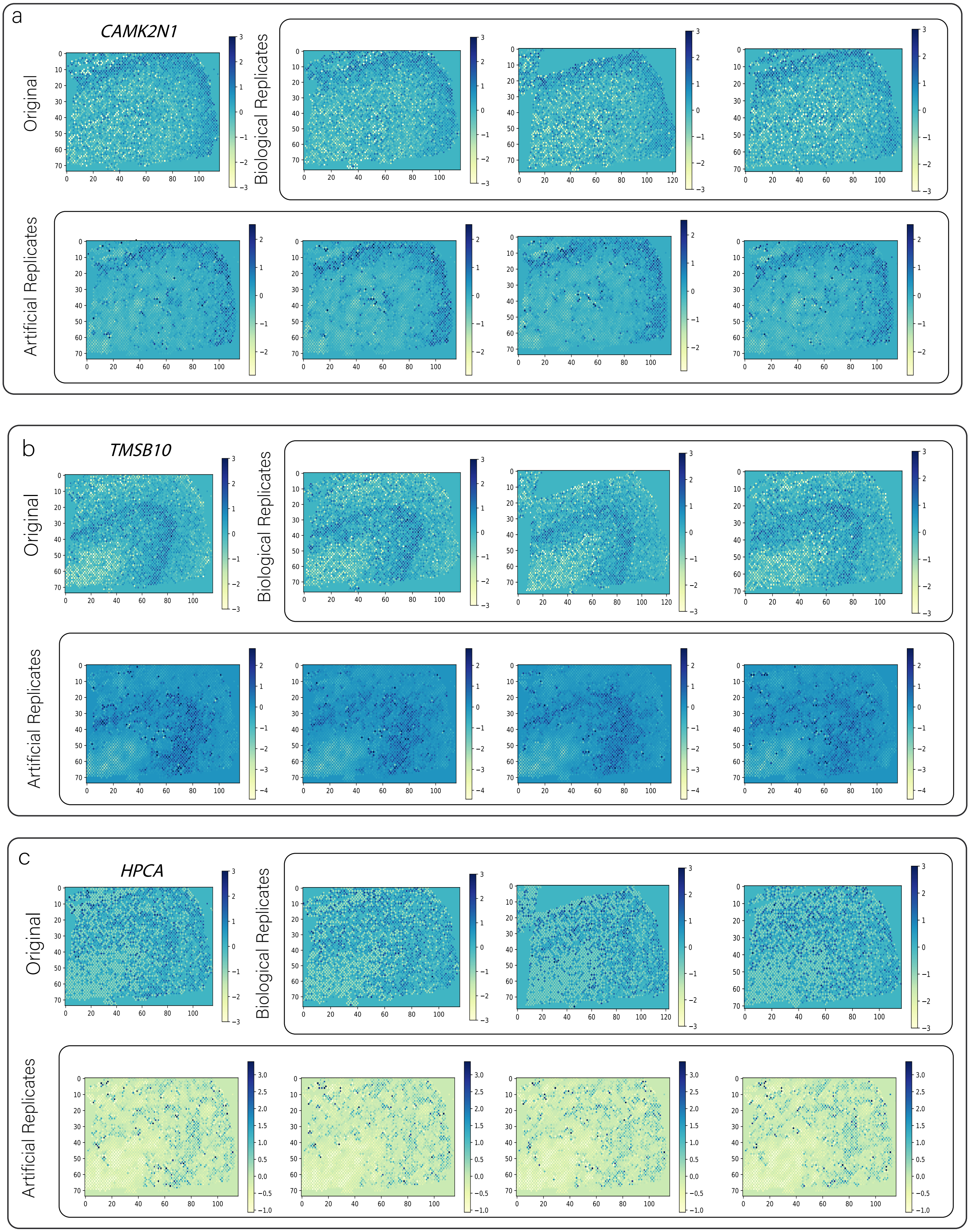

**Supplementary Fig. 4.** Additional results for the correlation of pairwise distances between true coordinates and the pairwise distances between predicted coordinates. (a) The additional results under the scenario of 10% and 50% holdoff, respectively. (b) Scatter density plots of pairwise distances of predicted locations versus those of the truth without resolution enhancement when the holdoff rate was 10%, 30%, and 50%, respectively.

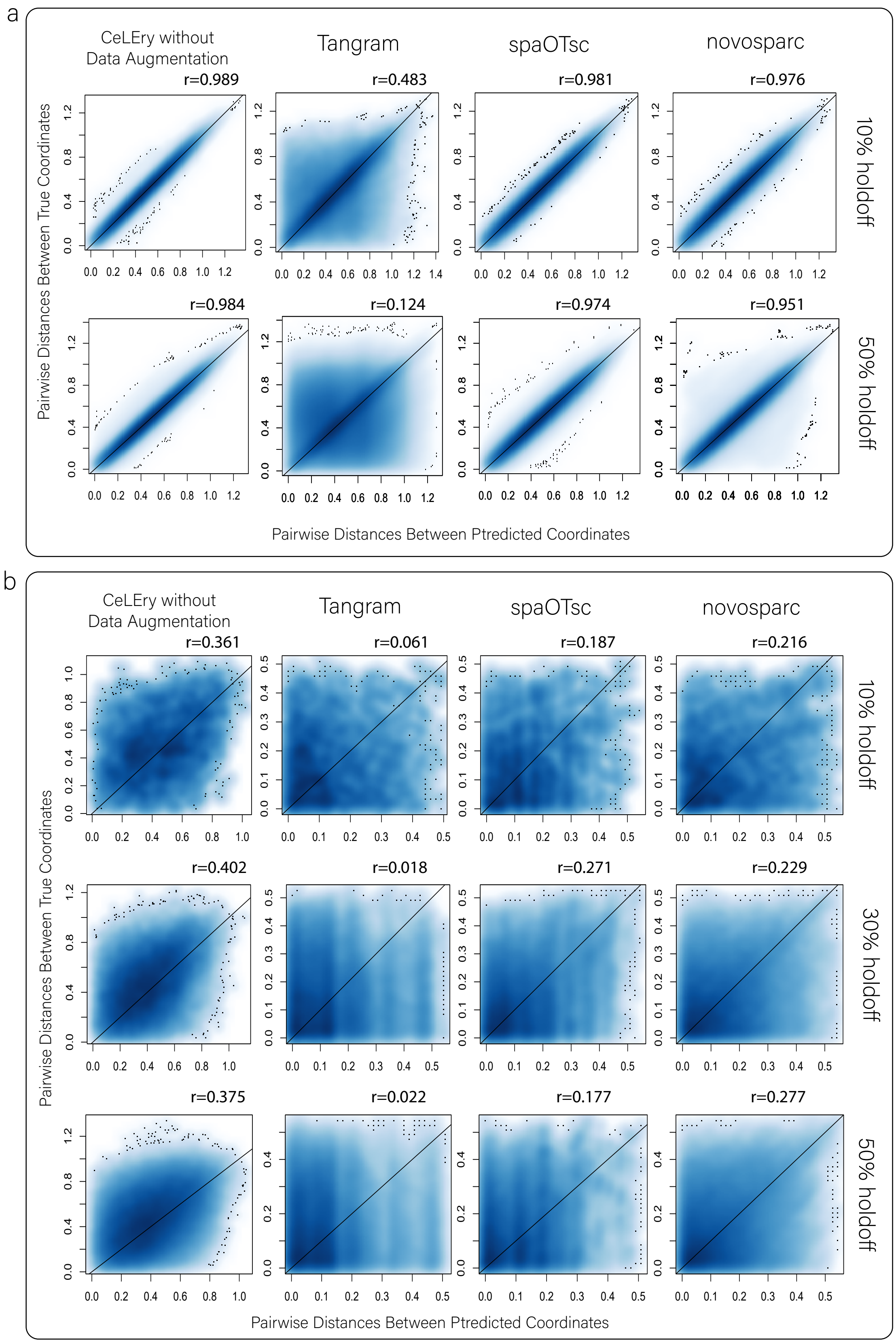

**Supplementary Fig. 5.** Boxplots of structural similarity index (SSIM) between predicted and true gene expression map in the mouse posterior brain dataset with different degrees of holdoff (*n* = 358 genes in each boxplot). The lower and upper hinges correspond to the first and third quartiles, and the center refers to the median value. The upper (lower) whiskers extend from the hinge to the largest (smallest) value no further (at most) than 1.5 × interquartile range from the hinge. Data beyond the end of the whiskers are plotted individually.

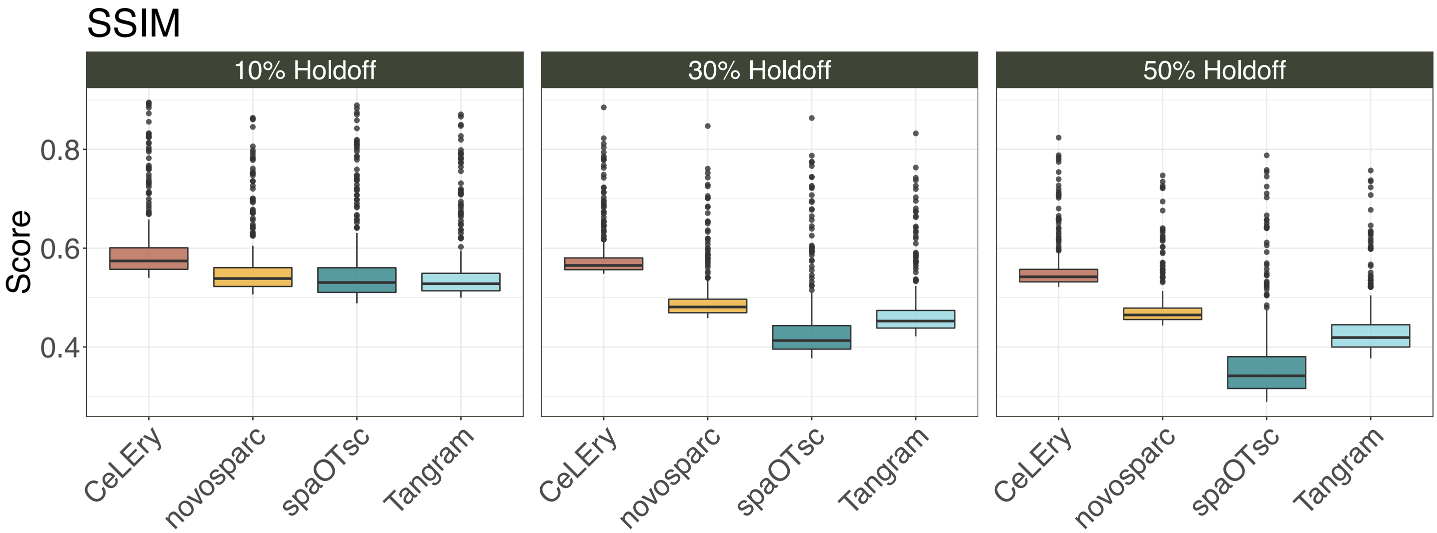

**Supplementary Fig. 6.** Heatmaps of the 2D location prediction certainty scores for spots in the test set (mouse posterior brain) when the confidence level was 0.5 (left) and 0.8 (right). The holdoff rate was 30%.

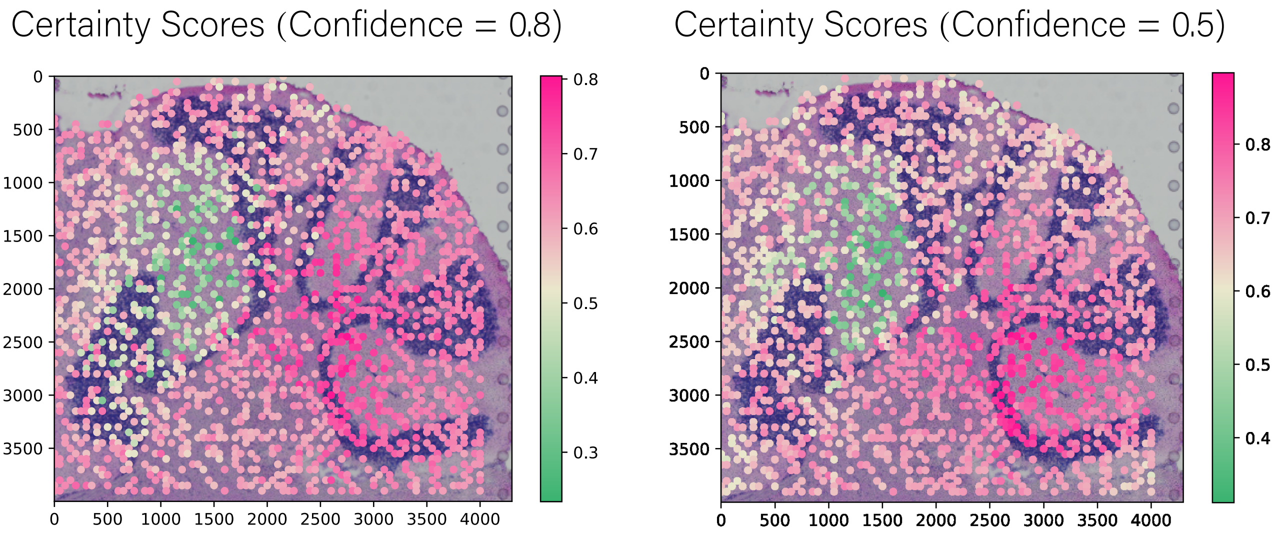

**Supplementary Fig. 7.** 2D location recovery for single cells in MERSCOPE mouse brain data. (a) The true cell location maps and predicted cell location maps based on locations predictd by CeLEry, Tangram, spaOTsc, and novoSpaRc for Scenario 1 shown in Fig. 6a. (b) Scatter density plot comparing true and predicted pairwise distances for all cells pairs in Scenario 1 and Scenario 2. Color in the plot indicates density of cell pairs. (c) Visualization of Euclidean distances between true and predicted locations for all cells in Scenario 1 and Scenario 2.

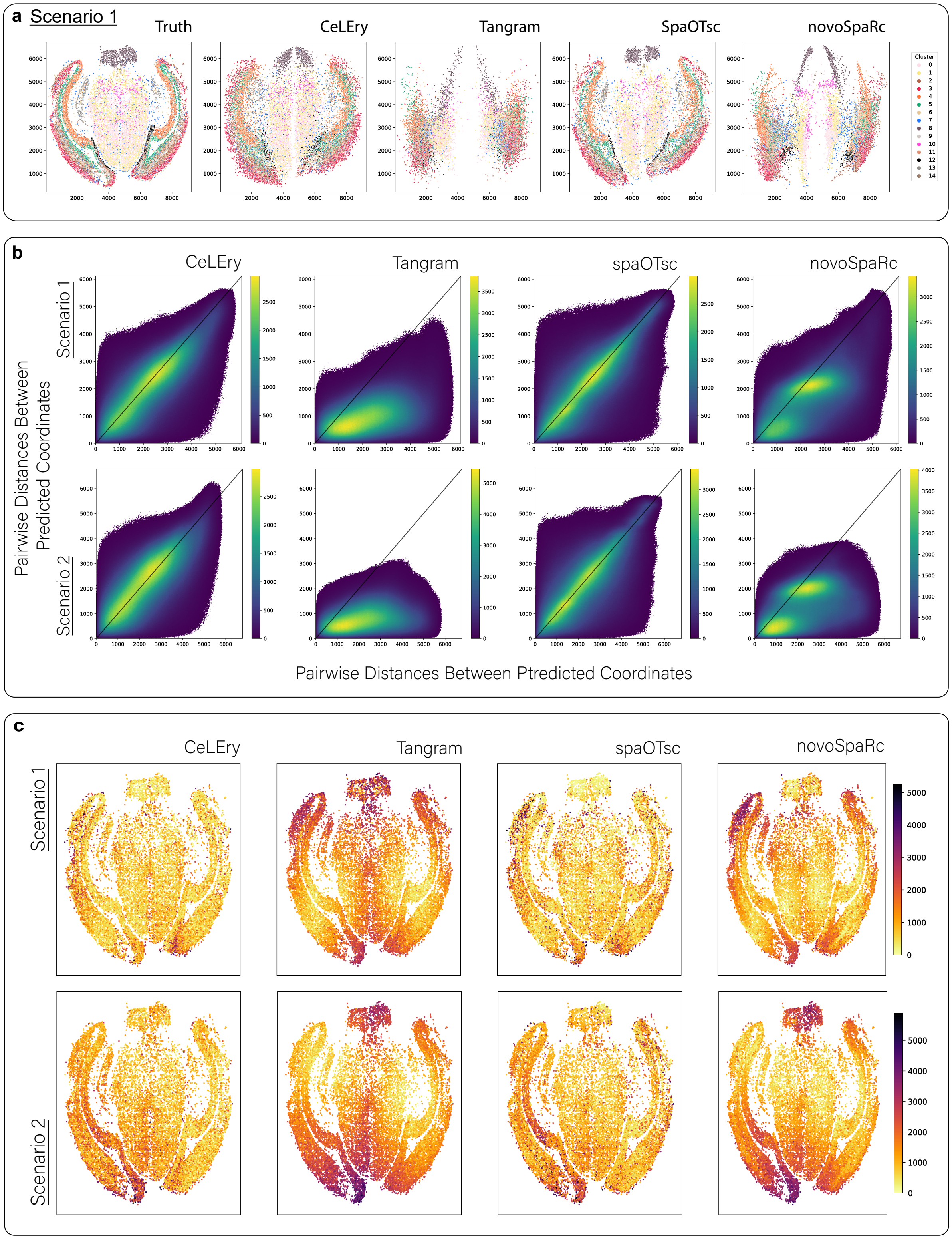

**Supplementary Fig. 8.** Recovered gene expression map of six randomly selected genes in the MERSCOPE mouse brain data, based on the predicted locations by CeLEry in Scenario 3 shown in Fig. 6a, with color indicating relative gene expression.

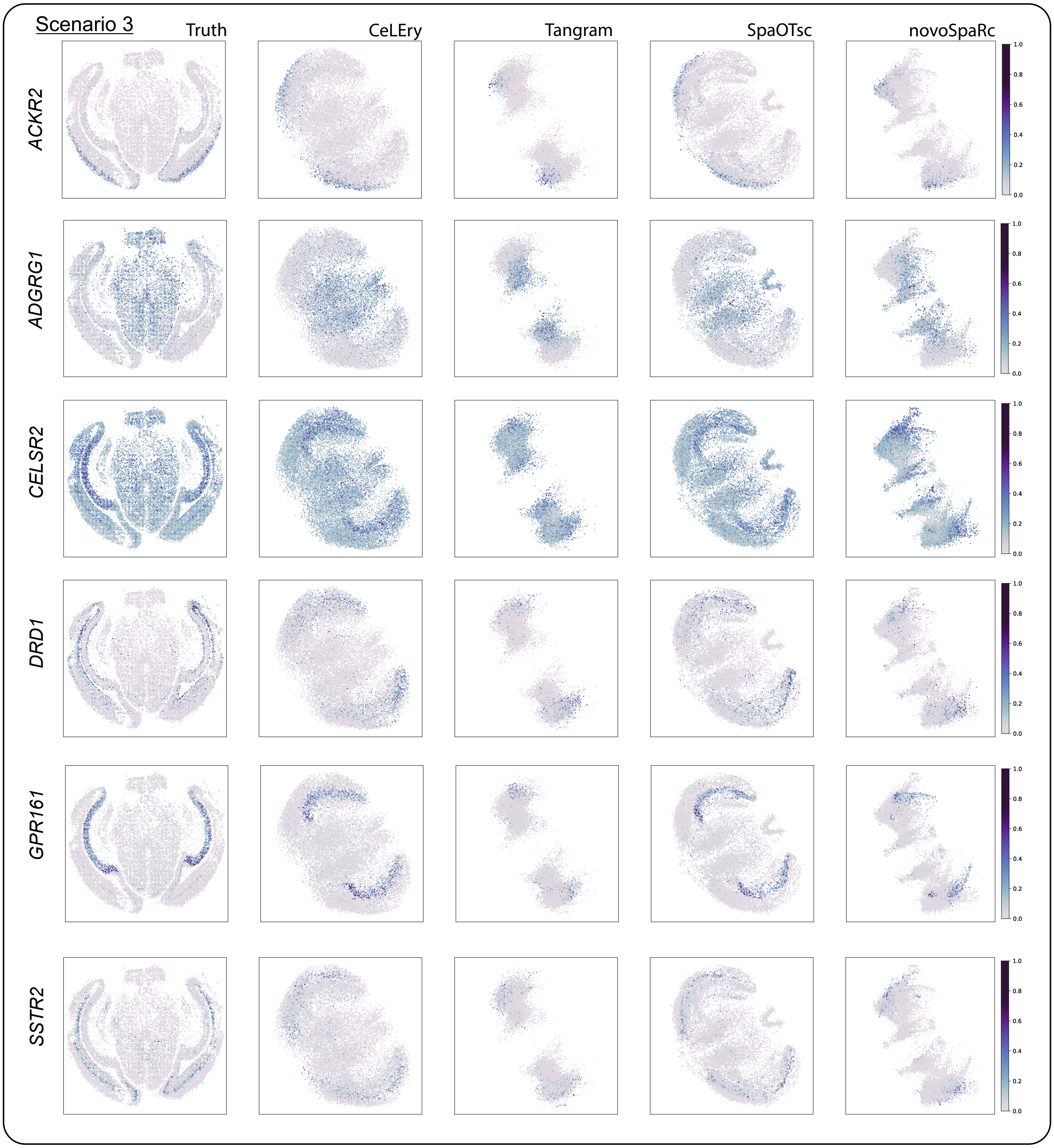

**Supplementary Fig. 9.** 2D location recovery for single cells in the MERFISH mouse brain data by Zhang et al. (a) Scatter density plot comparing true and predicted pairwise distances for all cells pairs in Scenario 1 shown in Fig. 7a. Color in the plot indicates density of cell pairs. (b) Visualization of Euclidean distances between true and predicted locations for all cells in Scenario 1. (c) True and recovered gene expression maps of additional ligand-receptor pairs, based on the predicted locations by CeLEry for Scenario 2, with color indicating relative gene expression.

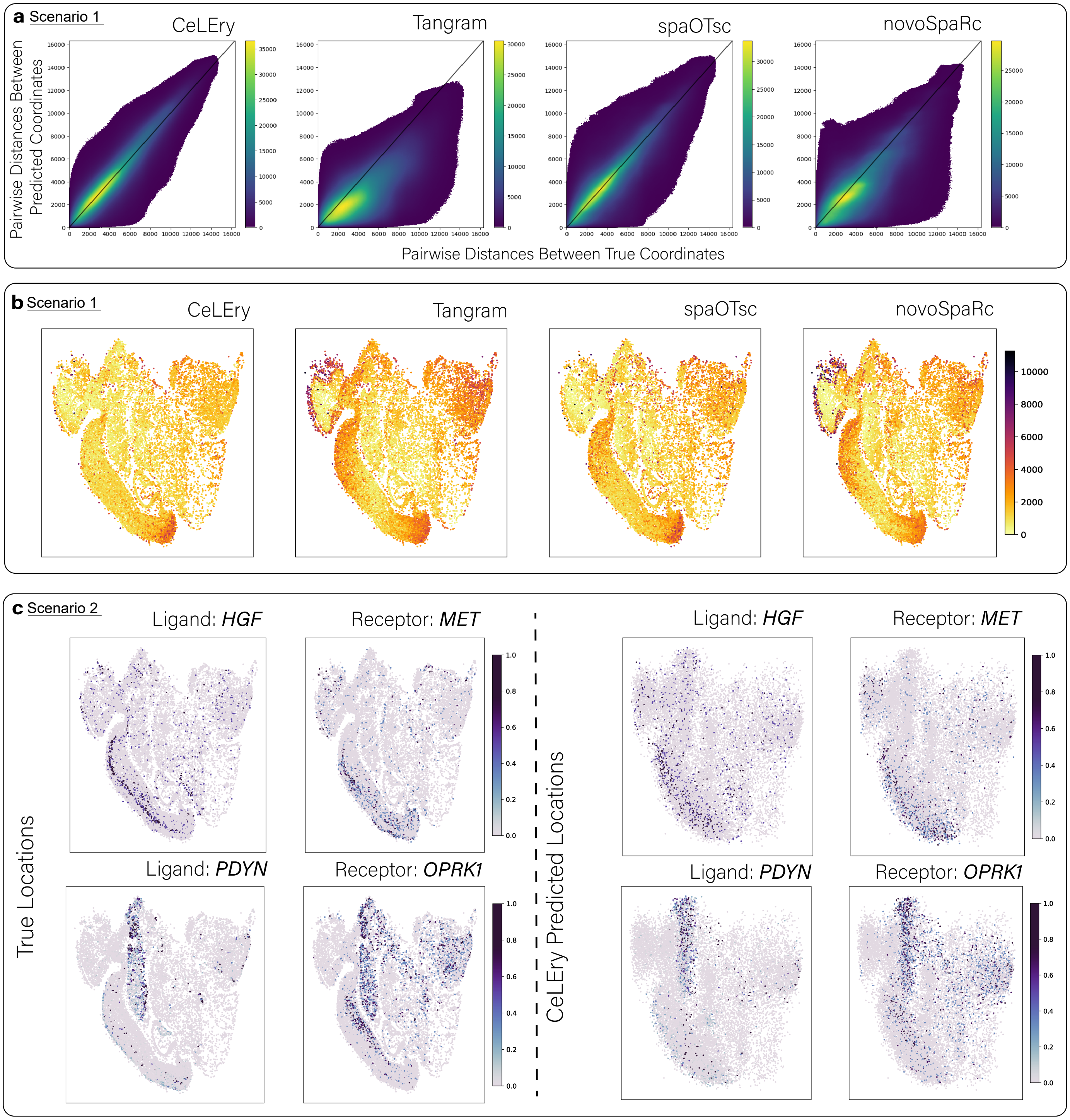

**Supplementary Fig. 10.** 2D location recovery for single cells in MERSCOPE human liver cancer data. (a) The true cell location maps and predicted cell location maps based on locations predictd by CeLEry, Tangram, spaOTsc, and novoSpaRc. (b) Recovered gene expression map of six randomly selected genes based on the predicted locations by CeLEry, with color indicating relative gene expression.

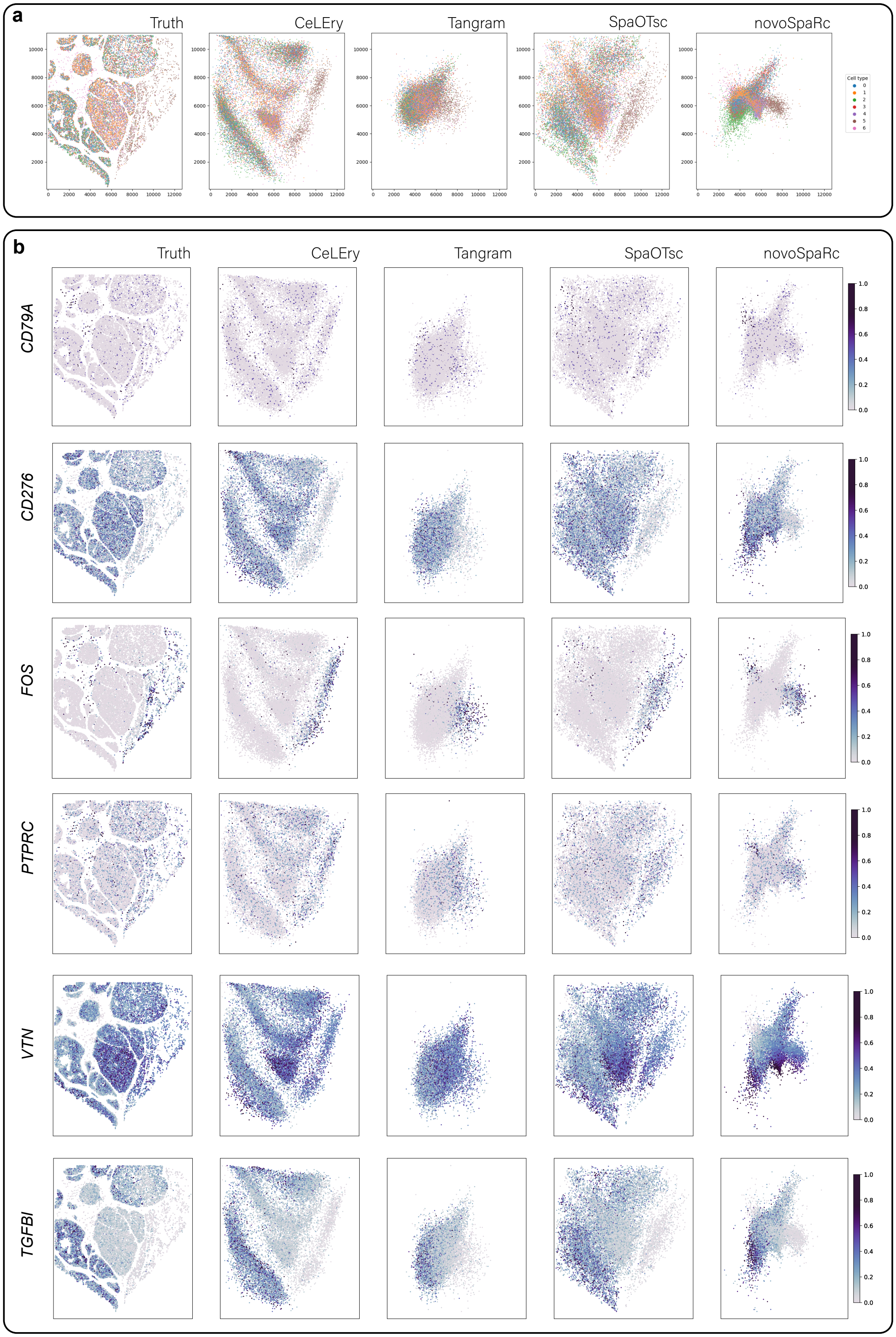

**Supplementary Fig. 11.** 2D location recovery for single cells in 10X Xenium breast cancer data for Scenario 1. (a) The true cell location maps and the predicted cell location maps generated by CeLEry, Tangram, spaOTsc, and novoSpaRc, with cells annotated according to graph-based cluster assignment and corresponding cell-type for the test cells in Replicate 2. (b) Scatter plots depicting the comparison between the true and predicted coordinates for both x-axis and y-axis in the 10x Xenium breast cancer dataset. Each column represents the results from a different method (CeLEry, Tangram, spaOTsc, and novoSpaRc) while each row shows the comparison in a different direction (x-axis and y-axis).

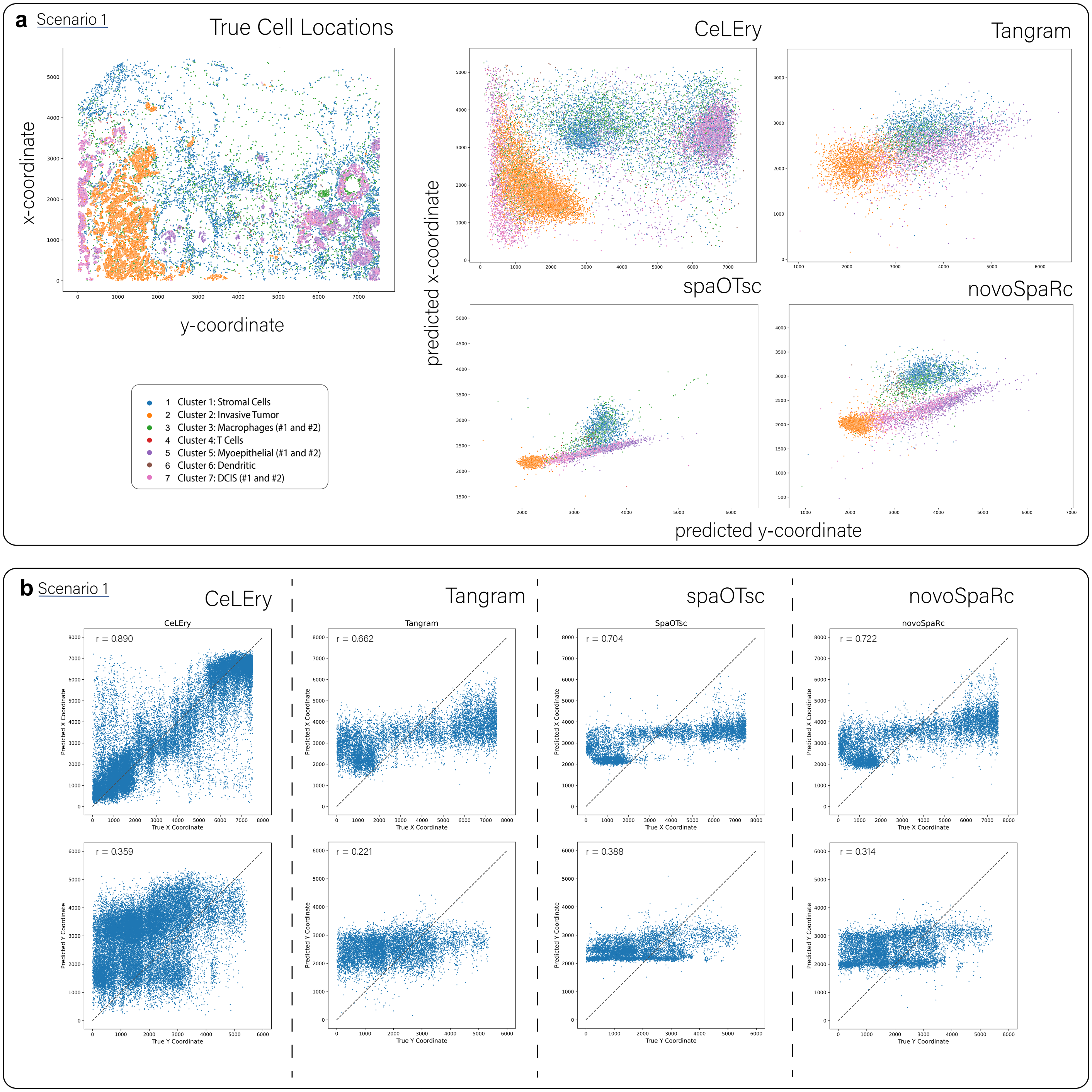

**Supplementary Fig. 12.** Further exploration of results for Scenarios 2 and 3 across methods for the Xenium breast cancer analysis. (a,c) Scatter density plots comparing true and predicted pairwise distances for all cell pairs in Scenarios 2 and 3 respectively. Color in the plots indicates density of cell pairs. (b,d) Visualization of Euclidean distances between true and predicted locations for all cells in the test data for Scenarios 2 and 3 respectively.

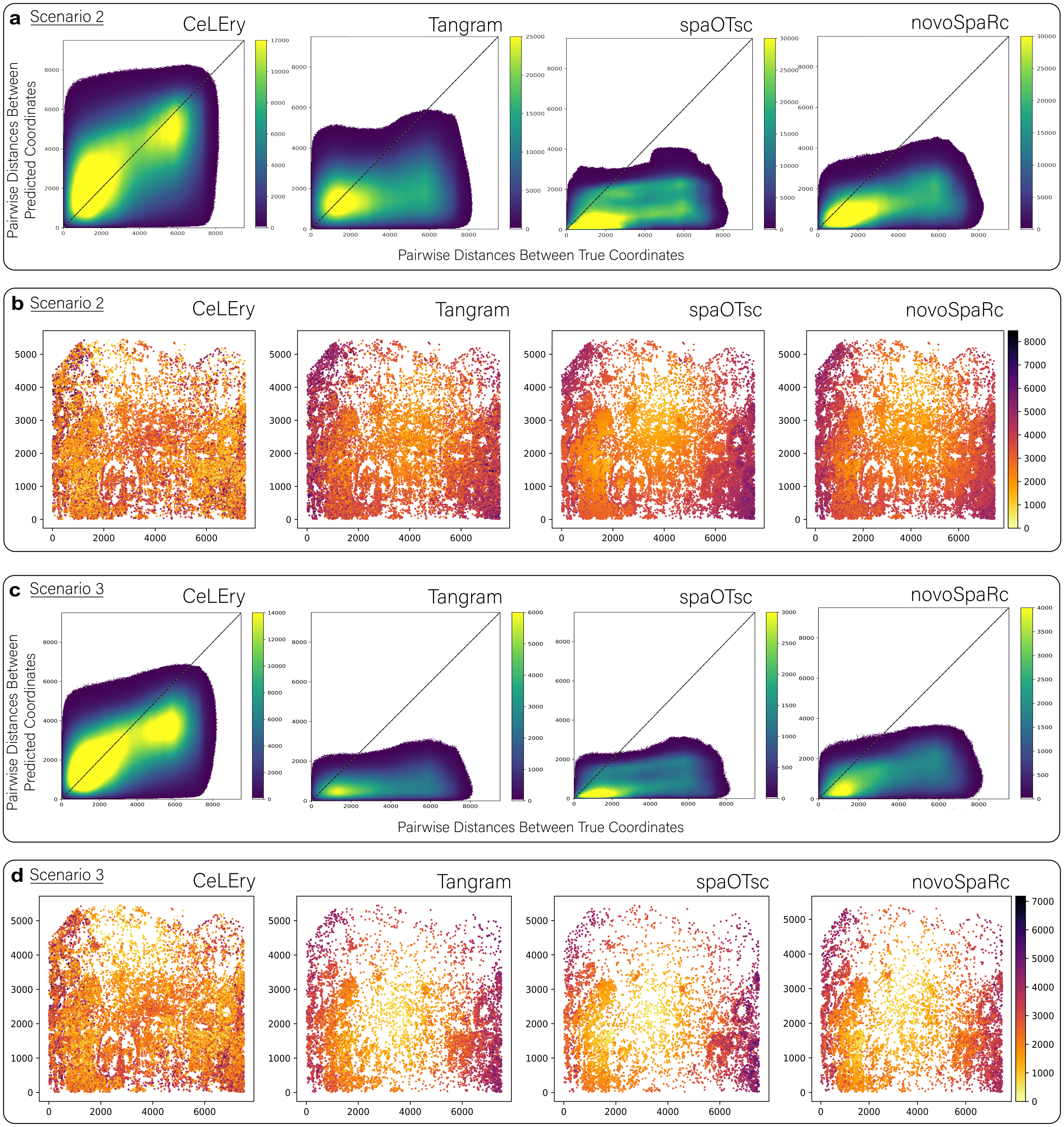

**Supplementary Fig. 13.** Additional recovered gene expression maps of four randomly selected genes along with two important breast cancer genes, *ERBB2* and *ESR1*, for single cells in the 10x Xenium breast cancer data, based on the predicted locations by each method for Replicate 2.

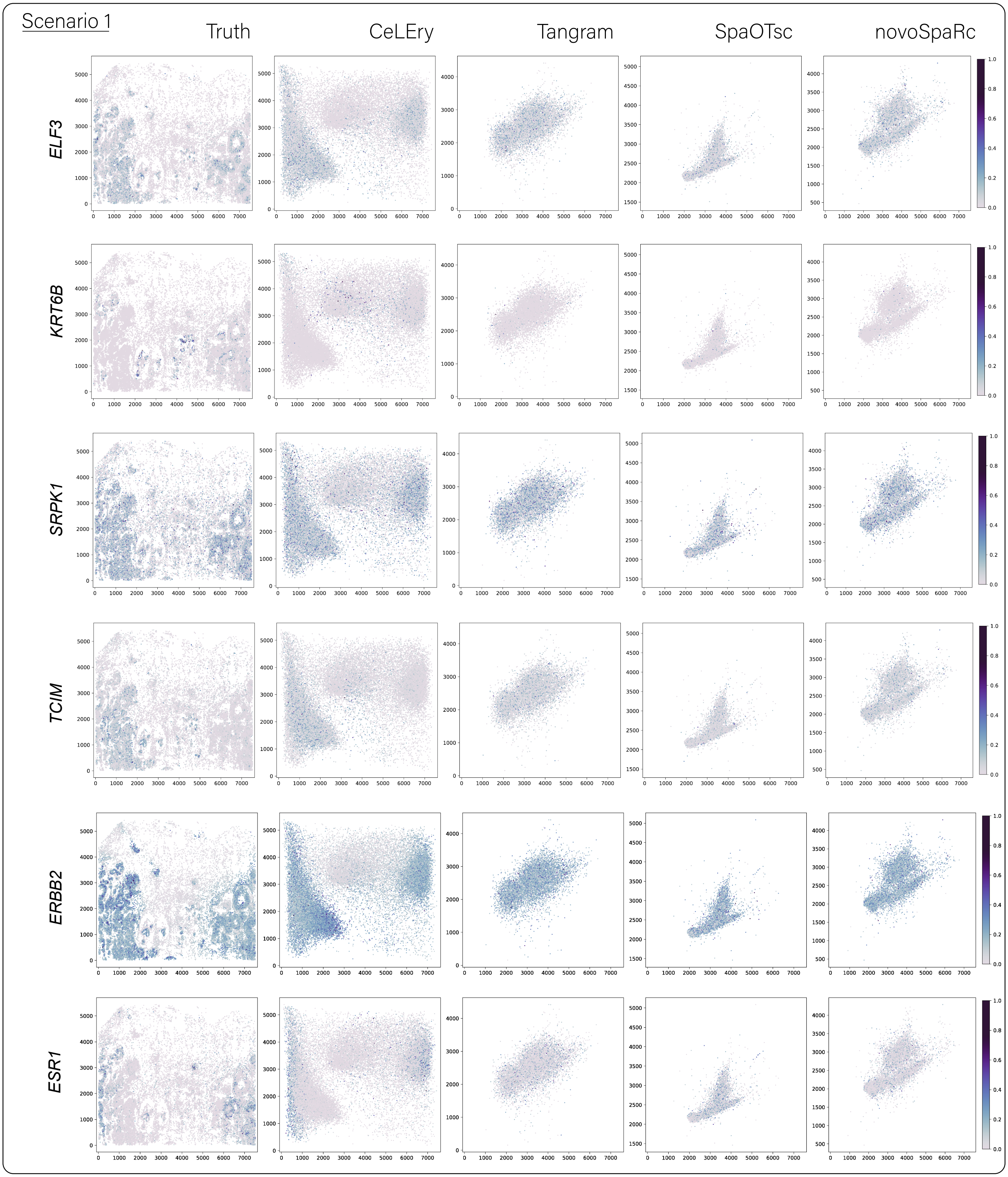

**Supplementary Fig. 14.** Cell type recovery for 10X Xenium breast cancer data in Replicate 1. (a) The true cell types of cells in breast cancer data, annotated by the cluster results of performing K-means (K=10). (b) The predicted cell types visualization generated by CeLEry, Tangram, SpaOTsc and novoSpaRc, whose overall prediction accuracies are 96.3%, 88.4%, 85.1% and 86.3%, respectively.

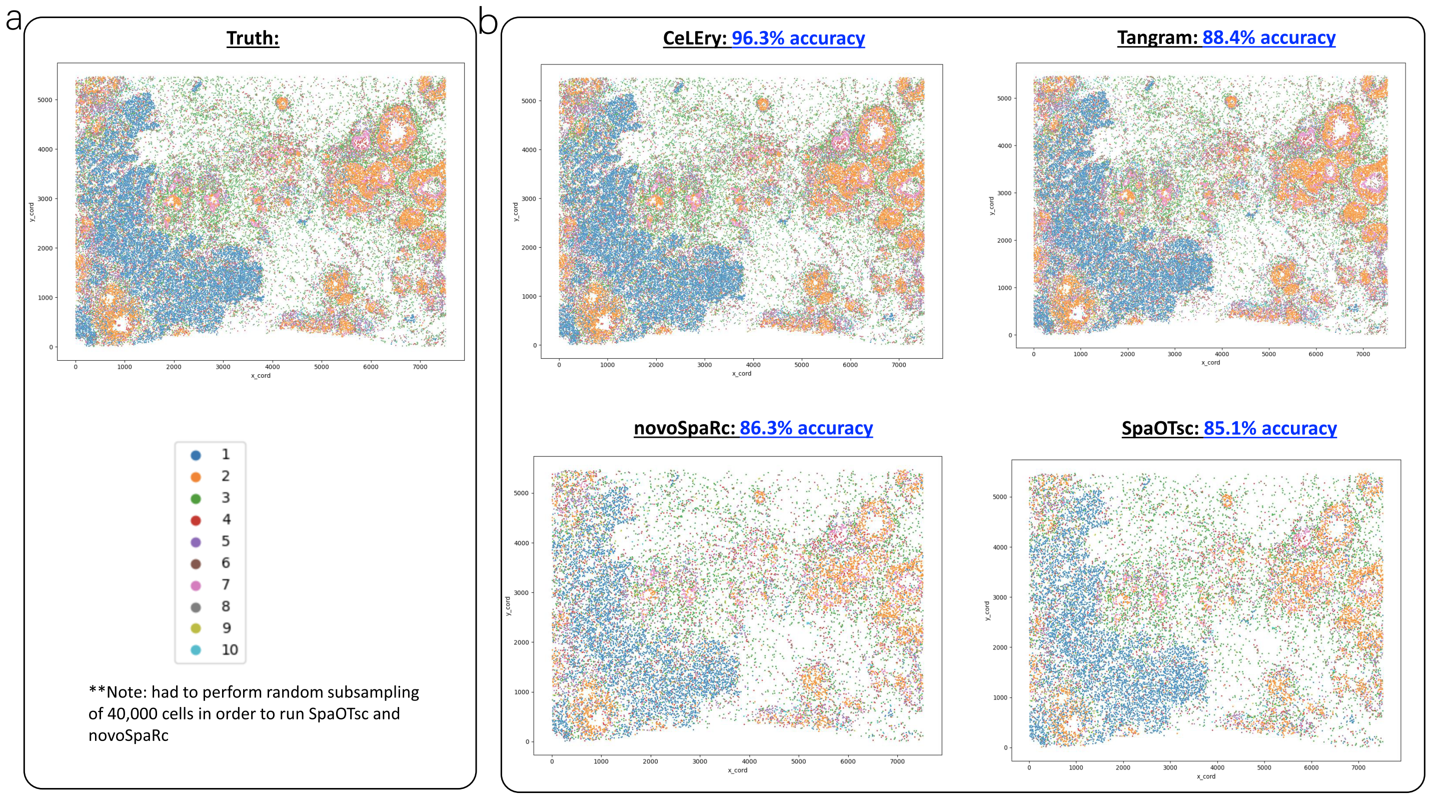

Supplement: Supplementary file 1 — Supplementary Information [file 41467_2023_39895_MOESM1_ESM.pdf]
