# Supplementary material for: Leveraging spatial transcriptomics data to recover cell locations in single-cell RNA-seq with CeLEry
Source: Nat Commun. 2023 Jul 8;14:4050. doi: 10.1038/s41467-023-39895-3 (PMC10329686; doi:10.1038/s41467-023-39895-3)
Supplement: Supplementary file 3 — Reporting Summary [file 41467_2023_39895_MOESM3_ESM.pdf]

Corresponding author(s): Qihuang Zhang, Mingyao Li

Last updated by author(s): Jun 9, 2023

Reporting Summary

Nature Portfolio wishes to improve the reproducibility of the work that we publish. This form provides structure for consistency and transparency in reporting. For further information on Nature Portfolio policies, see our Editorial Policies and the Editorial Policy Checklist.

Statistics

For all statistical analyses, confirm that the following items are present in the figure legend, table legend, main text, or Methods section.

n/a	Confirmed	
☐	☒	The exact sample size (*n*) for each experimental group/condition, given as a discrete number and unit of measurement
☐	☒	A statement on whether measurements were taken from distinct samples or whether the same sample was measured repeatedly
☐	☒	The statistical test(s) used AND whether they are one- or two-sided<br>*Only common tests should be described solely by name; describe more complex techniques in the Methods section.*
☐	☒	A description of all covariates tested
☒	☐	A description of any assumptions or corrections, such as tests of normality and adjustment for multiple comparisons
☐	☒	A full description of the statistical parameters including central tendency (e.g. means) or other basic estimates (e.g. regression coefficient) AND variation (e.g. standard deviation) or associated estimates of uncertainty (e.g. confidence intervals)
☐	☒	For null hypothesis testing, the test statistic (e.g. *F*, *t*, *r*) with confidence intervals, effect sizes, degrees of freedom and *P* value noted<br>*Give P values as exact values whenever suitable.*
☒	☐	For Bayesian analysis, information on the choice of priors and Markov chain Monte Carlo settings
☐	☒	For hierarchical and complex designs, identification of the appropriate level for tests and full reporting of outcomes
☐	☒	Estimates of effect sizes (e.g. Cohen's *d*, Pearson's *r*), indicating how they were calculated

Our web collection on statistics for biologists contains articles on many of the points above.

Software and code

Policy information about availability of computer code

Data collection	No software was used for data collection.
Data analysis	Data analyses were performed using Python 3.8 and R 4.0. CeLEry v1.1.2 (https://github.com/QihuangZhang/CeLEry), Tangram v1.0.4 (https://github.com/broadinstitute/Tangram), novoSpaRc v0.4.4 (https://github.com/rajewsky-lab/novosparc) and spaOTsc v0.2 (https://github.com/zcang/SpaOTsc) were used to conduct location recovery for scRNA-seq data. spaGCN v1.2.7 (https://github.com/jianhuupenn/SpaGCN) was used for tissue region segmentation. TESLA v1.2.4 (https://github.com/jianhuupenn/TESLA) was used to generate super resolution gene expression data. R package ggplot2 v3.4.2 is used for the visualization of the results.

For manuscripts utilizing custom algorithms or software that are central to the research but not yet described in published literature, software must be made available to editors and reviewers. We strongly encourage code deposition in a community repository (e.g. GitHub). See the Nature Portfolio guidelines for submitting code & software for further information.

Data

Policy information about availability of data

All manuscripts must include a data availability statement. This statement should provide the following information, where applicable:

- Accession codes, unique identifiers, or web links for publicly available datasets
- A description of any restrictions on data availability
- For clinical datasets or third party data, please ensure that the statement adheres to our policy

The ST and sc/snRA-seq data analyzed in this paper can be acquired from the following websites or accession numbers:
(1) LIBD human DLPFC 10x Visium data (http://research.libd.org/spatialLIBD/);
(2) mouse posterior brain 10x Visium data (https://support.10xgenomics.com/spatial-gene-expression/datasets/1.0.0/V1_Mouse_Brain_Sagittal_Posterior);
(3) AD snRNA-seq data (https://upenn.app.box.com/s/e8nf4b384s7oi3o09pj5s8jfdu11swim);
(4) mouse brain MERSCOPE data (https://info.vizgen.com/mouse-brain-data);
(5) Mouse Brain MERFISH data (https://doi.org/10.35077/act-bag);
(6) Mouse Whole Cortex and Hippocampus 10x scRNA-seq data used to predict the 2D locations onto the mouse brain reference data (https://portal.brain-map.org/atlases-and-data/rnaseq/mouse-whole-cortex-and-hippocampus-10x);
(7) Human liver cancer MERSCOPE data (https://console.cloud.google.com/storage/browser/vz-ffpe-showcase/HumanLiverCancerPatient2);
(8) 10X Xenium data (https://www.10xgenomics.com/welcome?closeUrl=%2F&lastTouchOfferName=Xenium+Preprint+Dataset&lastTouchOfferType=Dataset&product=xenium&redirectUrl=%2Fproducts%2Fxenium-in-situ%2Fpreview-dataset-human-breast).

Human research participants

Policy information about studies involving human research participants and Sex and Gender in Research.

Reporting on sex and gender	N/A
Population characteristics	N/A
Recruitment	N/A
Ethics oversight	N/A

Note that full information on the approval of the study protocol must also be provided in the manuscript.

Field-specific reporting

Please select the one below that is the best fit for your research. If you are not sure, read the appropriate sections before making your selection.

☒ Life sciences ☐ Behavioural & social sciences ☐ Ecological, evolutionary & environmental sciences

For a reference copy of the document with all sections, see nature.com/documents/nr-reporting-summary-flat.pdf

Life sciences study design

All studies must disclose on these points even when the disclosure is negative.

Sample size	Sample size in our study is the number of cells in single-cell RNA-seq or the number of spots in spatial transcriptomics data. The number of cells or spots is determined by the original study in which the data were downloaded from.
Data exclusions	Highly variable genes or top differentially expressed genes between spatial domains were selected when training the cell location prediction model. In the study of MERSCOPE, MERFISH, and 10X Xenium data, we excluded cells with low UMI counts or small number of expressed genes.
Replication	We trained the CeLEry model based on multiple datasets (including 2 10X visium data, 2 MERSCOPE data, 1 MERFISH data, and 1 10X Xenium data) in benchmark studies and applied the trained model to different query single-cell RNA-seq data (1 Alzheimer's disease snRNA-seq data and 1 mouse whole cortex scRNA-seq data). Our conclusions are consistent from these replications, which demonstrated that our proposed method is robust in predicting cell locations in single-cell RNA-seq.
Randomization	No randomization was conducted in our study. The primary objective of our research is to predict cell locations, which does not involve statistical modeling with covariates. Moreover, our predictive modeling does not involve assigning treatments. As a result, randomization is unnecessary for our study.
Blinding	The main objective of our study is to predict cell locations in single-cell RNA-seq data by utilizing gene expression information. As our study does not involve analyzing treatment effects, there is no need for blinding.

Reporting for specific materials, systems and methods

We require information from authors about some types of materials, experimental systems and methods used in many studies. Here, indicate whether each material, system or method listed is relevant to your study. If you are not sure if a list item applies to your research, read the appropriate section before selecting a response.

Materials & experimental systems

n/a	Involved in the study
☒	☐ Antibodies
☒	☐ Eukaryotic cell lines
☒	☐ Palaeontology and archaeology
☒	☐ Animals and other organisms
☒	☐ Clinical data
☒	☐ Dual use research of concern

Methods

n/a	Involved in the study
☒	☐ ChIP-seq
☒	☐ Flow cytometry
☒	☐ MRI-based neuroimaging
